# *Fasciola hepatica*: Histology of the Reproductive Organs and Differential Effects of Triclabendazole on Drug-Sensitive and Drug-Resistant Fluke Isolates and on Flukes from Selected Field Cases

**DOI:** 10.3390/pathogens4030431

**Published:** 2015-06-26

**Authors:** Robert Hanna

**Affiliations:** Section of Parasitology, Disease Surveillance and Investigation Branch, Veterinary Sciences Division, Agri-Food and Biosciences Institute, Stormont, Belfast BT4 3SD, UK; E-Mail: bob.hanna@afbini.gov.uk; Tel.: +44-2890-525615

**Keywords:** *Fasciola hepatica*, sheep, cattle, triclabendazole-sensitive, triclabendazole-resistant, fluke morphology, fluke histology, fluke reproductive organs, *in situ* hybridization, apoptosis

## Abstract

This review summarises the findings of a series of studies in which the histological changes, induced in the reproductive system of *Fasciola hepatica* following treatment of the ovine host with the anthelmintic triclabendazole (TCBZ), were examined. A detailed description of the normal macroscopic arrangement and histological features of the testes, ovary, vitelline tissue, Mehlis’ gland and uterus is provided to aid recognition of the drug-induced lesions, and to provide a basic model to inform similar toxicological studies on *F. hepatica* in the future. The production of spermatozoa and egg components represents the main energy consuming activity of the adult fluke. Thus the reproductive organs, with their high turnover of cells and secretory products, are uniquely sensitive to metabolic inhibition and sub-cellular disorganisation induced by extraneous toxic compounds. The flukes chosen for study were derived from TCBZ-sensitive (TCBZ-S) and TCBZ-resistant (TCBZ-R) isolates, the status of which had previously been proven in controlled clinical trials. For comparison, flukes collected from flocks where TCBZ resistance had been diagnosed by coprological methods, and from a dairy farm with no history of TCBZ use, were also examined. The macroscopic arrangement of the reproductive system in flukes was studied using catechol/carmine stained whole mounts, and the histology of the main organs was examined using conventional haematoxylin-eosin stained sections. Validation of apoptosis in the fluke sections was carried out using an *in situ* hybridisation method designed to label endonuclease-induced DNA strand breaks. In TCBZ-S flukes exposed to TCBZ metabolites for 24–96 h *in vivo*, but not in TCBZ-R flukes, those tissues where active meiosis and/or mitosis occurred (testis, ovary, and vitelline follicles), were found to display progressive loss of cell content. This was due to apparent failure of cell division to keep pace with expulsion of the mature or effete products. Further, actively dividing cell types tended to become individualised, rounded and condensed, characteristic of apoptotic cell death. In the treated TCBZ-S flukes, strong positive labelling indicating apoptosis was associated with the morphologically abnormal cells undergoing mitosis or meiosis in the testis, ovary and vitelline follicles. In treated flukes from field outbreaks of suspected TCBZ-R fasciolosis, no significant histological changes were observed, nor was there any positive labelling for apotosis. On the other hand, sections of TCBZ treated flukes derived from a field case of fasciolosis where TCBZ resistance was not suspected displayed severe histological lesions, and heavy positive labelling for apoptosis. The triggering of apoptosis is considered to be related to failure of spindle formation at cell division, supporting the contention that TCBZ inhibits microtubule formation. In treated TCBZ-S flukes, protein synthesis and transport was apparently inhibited in the Mehlis’ secretory cells, perhaps due to energy uncoupling or to microtubule defects. In the uterus, successful formation of shelled eggs represents the culmination of a complex sequence of cytokinetic, cytological and synthetic activity involving the vitelline follicles, the ovary and the Mehlis’ gland. Histological evidence indicating failure of ovigenesis in TCBZ-S flukes was evident from as early as 24 h post-treatment onwards. Light labelling for apoptosis was associated with the testis of untreated Cullompton (TCBZ-S) and Sligo type 2 (TCBZ-R) flukes, which exhibit abnormal spermatogenesis and spermiogenesis, respectively. This was attributed to apoptosis and to heterophagy of effete germ line cells by the sustentacular tissue. The studies summarised in this review illustrate the potential utility of histological techniques for conveniently screening representative samples of flukes in field trials designed to validate instances of drug resistance. Histology can also be used to test the efficacy of new products against known drug-resistant and drug-susceptible fluke isolates. The account also provides reference criteria for drug-induced histopathological changes in fluke reproductive structures, examination of which may supplement and augment conventional coprological testing, and aid interpretation of TEM findings.

## 1. Introduction

The life cycle of the common liver fluke, *Fasciola hepatica* was elucidated by Leuckart (1882) [1] and Lutz (1892, 1893) [2,3] and the details have recently been reviewed by Andrews (1999) [4]. As a typical digenetic trematode, the adult fluke is hermaphroditic, exhibiting two highly branched testes located one behind the other in the posterior half of the body, and a single dendritic ovary, located on the left of the uterus, as viewed from the ventral surface. Extensively developed vitelline glands that provide the precursor shell-proteins and glycogen stores needed for egg production are located lateral and posterior to the testes. The arrangement of the gonads, accessory reproductive organs and ducts within the body of the fluke, and in particular around the ootype where the eggs are assembled, is readily visualised using carmine-stained whole mount preparations. Such preparations can provide useful information on the overall state of reproductive development in fluke populations under the influence of anthelmintics administered to the host [5,6,7].

With an impressively high level of egg production, reaching 25,000 per fluke per day [8], and with a large proportion of the body occupied by testis and vitelline tissue, it is evident that the production of spermatozoa and egg components represents the main energy consuming activity of the adult fluke [5]. While it has been recognised that single flukes in a definitive host animal are capable of producing eggs, and hence that self-fertilization is possible [9], it has been generally assumed that cross-fertilization is the preferred reproductive strategy when a number of individuals occur together in a single host liver. Recent studies on single-fluke infections in rats indicate that self-fertilization may occur in only about 33% of flukes without a co-inhabitant, suggesting that parthenogenesis is a possible reproductive strategy [10]. This is supported by the fact that flukes of the triploid aspermic Cullompton isolate are highly fecund, and the eggs they produce develop and hatch normally, giving rise to viable miracidia. These are capable of infecting the molluscan intermediate host and perpetuating this clonal isolate through subsequent generations [11].

Scanning and transmission electron microscopy have often been used to help elucidate the effects of anthelmintic drugs on specific tissues of liver flukes [12]. However, conventional histopathological methods (including whole-mounts) have been little used until recently, despite their potential value in screening simultaneously all tissues in statistically viable samples of flukes exposed to anthelmintic drugs *in vivo* or *in vitro*. A primary aim of this report is to clarify aspects of the histology of the reproductive system in untreated flukes and thus provide a basis for characterising the action of anthelmintic drugs by their histological and morphological effects on the reproductive system. The latter, due to the high metabolic demands of sperm and egg production, is uniquely sensitive to adverse physiochemical and pharmacological conditions. Examination of the histological and morphological changes induced in the reproductive system of flukes after treatment of their host with anthelmintics such as triclabendazole (TCBZ) can provide useful information on the drug-resistance status of individual fluke isolates.

TCBZ, a benzimidazole anthelmintic with fasciolicidal activity, was introduced as a veterinary drug in 1983, and is active against *Fasciola hepatica*, *Fasciola gigantica*, *Fascioloides magna* and *Paragonimus* spp., but not against nematodes or cestodes [13]. Its importance and widespread use derive mainly from the fact that it kills flukes from as early as two days post-infection through to the fully mature adult stage, thus offering effective control for both acute and chronic fasciolosis [12,14]. Over-reliance on this drug has, however, led to the development of resistance to TCBZ in fluke populations worldwide, the first case having been reported from Australia in 1995 [15]. Since then resistance has been reported throughout Europe, including cases from the British Isles [13]. Previous studies [12,16] have shown that TCBZ, like other anthelmintic molecules in the benzimidazole class, may act as a β-tubulin antagonist. These molecules are believed to interfere with the assembly of microtubules which have essential roles in the movement of subcellular components and metabolites within the cytoplasm, as well as spindle formation during cell division.

Studies on the efficacy of TCBZ have centered mainly on field trials involving pre- and post-treatment fluke counts, faecal egg count reduction (FECRT) and clinical chemistry. Qualitative assessment has been carried out on ultrastructural lesions induced in flukes maintained under *in vitro* conditions in the presence of anthelmintic metabolites [17,18,19,20,21] or exposed *in vivo* in anthelmintic-treated sheep [6,22,23,24,25,26,27]. The TCBZ resistance status of populations of *F. hepatica* in field cases of fasciolosis, where treatment failure has been reported, is generally investigated by examination of faecal samples collected pre- and three weeks post-treatment with TCBZ. Tests such as FECRT and coproantigen reduction (CRT) are used [28,29], although these approaches are ineffective for diagnosis of the pre-bile duct stages of fasciolosis.

Histological examination of flukes following *in vivo* exposure to anthelmintics such as TCBZ offers the possibility of conveniently screening large numbers of flukes, or entire fluke populations from individual experimental or field infections. It can yield quantitative data relating to each and every parasite tissue. Such information can supplement and augment conventional coprological testing and aid or direct electron microscopy and stereology, which can be time-consuming, expensive and restricted in terms of numbers of fluke examined and range of tissues sampled in individual flukes. A histological approach was recently used to complement FECRT and CRT in a comparative study of the relative efficacies of TCBZ, nitroxynil and closantel in the control of fasciolosis on sheep farms in Northern Ireland [30].

## 2. Methods and Materials

### 2.1. Sources of Flukes

#### 2.1.1. Experimental Isolates

Ten shed-reared Blackface X sheep (4–5 months old), checked for the absence of helminth infection by faecal examination, were subsequently infected by oral gavage with 250 metacercariae obtained from laboratory colonies of *Galba truncatula* maintained at Queen’s University, Belfast, Northern Ireland. Six of the sheep received metacercariae of the TCBZ-sensitive (TCBZ-S) Cullompton fluke isolate and four received metacercariae of the TCBZ-resistant (TCBZ-R) Sligo fluke isolate. The TCBZ resistance status of these isolates was verified statistically in a series of experimental trials involving a total of 88 sheep [6,28,29,31,32]. Twelve weeks after infection, four of the TCBZ-S infected sheep were dosed with10 mg/kg TCBZ [33]. These sheep were slaughtered humanely by exsanguination following captive bolt stunning 24 h, 48 h, 72 h or 96 h after treatment, the livers were removed and all flukes present in the bile ducts and gall bladders were collected in warm (37 °C) 0.9% (w/v) NaCl. The remaining two TCBZ-S infected sheep were not treated with anthelmintic, but were slaughtered 24 h after the other sheep had been dosed, and the flukes from their livers were collected to provide untreated control material. Of the four sheep that were infected with the TCBZ-R fluke isolate, two were dosed with TCBZ (10 mg/kg; [33]) and slaughtered 48 h later for fluke collection. The remaining two sheep were not treated with anthelmintic, but were slaughtered 24 h after the other sheep were dosed, to provide untreated flukes for control material. The rational of dose-to-slaughter time was discussed by Hanna *et al*. (2010) [31]. The flukes on which the histological descriptions presented here were based were collected from sheep that formed part of a larger clinical trial, described by McConville *et al*. (2009) [6]. In this trial, the control group of untreated sheep that had been infected with the TCBZ-S isolate harboured, at autopsy, 142 ± 13 flukes, representing 57% infection success. The control group of untreated sheep infected with the TCBZ-R isolate were found to harbor 61 ± 8 flukes (25% infection success), while the treated group infected with the TCBZ-R isolate harboured 73 ± 8 flukes (29% infection success).

In Table 1, the infection, treatment and time-to-slaughter are summarized for those sheep involved in the work reported here. 

**Table 1:** Summary of the infection, treatment and post-treatment time to slaughter for the sheep from which flukes were collected for the purposes of the study reported here.

*F. hepatica* Infection	Number of Sheep Treated with 10 mg/kg TCBZ	Number of Sheep Receiving no Treatment	Time Post-Treatment at Which Flukes were Collected:			
			24 h	48 h	72 h	96 h
TCBZ-S Cullompton 250 mc (6 sheep)	4	2	1	1	1	1
			2			
TCBZ-R Sligo 250 mc (4 sheep)	2	2		2		
			2			

TCBZ-S = triclabendazole-sensitive isolate; TCBZ-R = triclabendazole-resistant isolate; mc = metacercariae.

#### 2.1.2. Field Cases with Suspected TCBZ- Resistance

Rectal faeces samples were collected individually from 10 sheep assembled on each of three widely-separated farms in Northern Ireland where TCBZ resistance was independently suspected (on the basis of previous treatment failure). These animals were individually dosed to weight with TCBZ (10 mg/kg). Seventy-two h later, on confirmation of a positive pre-treatment FEC and coproantigen result (using the protocols described by Flanagan *et al*., 2011 [28,29]), one animal from each group was slaughtered humanely by exsanguination following captive bolt stunning. The liver was removed and all flukes present in the bile ducts and gall bladder were collected in warm (37 °C) 0.9% (w/v) NaCl. In order to confirm failure of TCBZ treatment on each of the farms, rectal faeces samples were collected from the sheep remaining in each group three weeks after TCBZ treatment, and post-treatment FECs and coproantigen levels were determined. No significant differences was found, in any of the groups, between pre-treatment and 3-week post-treatment FECs and coproantigen levels, providing evidence of the TCBZ-resistance status of the fluke populations on each of the three farms [30]. The decision to collect flukes for histological examination from sheep dosed with TCBZ 72 h previously was based on the findings of time-course trials [31,34,35,36] which indicated that TCBZ-induced lesions in the reproductive organs of TCBZ-S flukes were very well developed by 72 h post-treatment, while sufficient flukes still remained in the bile ducts of sheep to enable sampling.

#### 2.1.3. Rats Infected with Metacercariae from Bovine Field Case

Approximately 150 specimens of *Galba truncatula* were collected from wet pasture on a dairy farm where fasciolosis had been diagnosed in the cattle in each of several successive years, but triclabendazole had not been used. The TCBZ-S status of the liver fluke population on this farm was confirmed by an appropriately-controlled dose-response trial using 30 rats experimentally infected with five metacercariae 12 weeks before treatment with 10 mg/kg TCBZ. At autopsy three weeks after treatment, no flukes were found in the bile ducts of the treated rats, in contrast to untreated control animals, each of which contained 2–5 flukes. The 3-week post-treatment faecal samples from the treated rats were found to be negative for fluke eggs. The use of rats as host animals, instead of sheep, as in the trials described above, reflected the very limited numbers of metacercariae available for use in the infections.

Snails from this dairy farm were induced to shed metacercariae, by immersing them in clean water chilled to 10 °C. Five metacercariae were delivered by oral gavage to each of a group of six male Wistar rats. Twelve weeks after infection, the rats were dosed orally with 10 mg/kg TCBZ, and 48 h later they were euthanized using CO2, prior to collection of flukes from the main bile duct of each animal. The decision to collect flukes from rats dosed with TCBZ 48 h previously was based on the findings of time-course trials [31,34,35,36] which showed that TCBZ-induced lesions in the reproductive organs of TCBZ-S flukes were readily identifiable 48 h post-treatment. Considering the low numbers of flukes present in the infected rats, it is likely that too few TCBZ-S flukes would have remained after 48 h to ensure sufficient numbers for sampling.

### 2.2. Preparation of Flukes for Histology

Fifteen flukes collected from each sheep, and all the flukes from the infected and treated rats were fixed for histological examination less than 30 min after removal from the host. The choice of a sample number of 15 flukes, collected for histology from each sheep, was dictated by the minimum number of flukes remaining in the TCBZ-S infected and treated sheep in the clinical trial (Section 2.1.1). It was also influenced by the fact that little histological variation was found within the sample groups of: (a) treated-susceptible; (b) untreated-susceptible; (c) treated-resistant; and (d) untreated-resistant flukes. The flukes were placed in a 10 cm-square plastic Petri dish and held flat beneath a light glass plate throughout fixation for 24 h with 10% (v/v) neutral-buffered formalin. After fixation, each fluke was sliced into equal right and left halves along the median plane. The two halves were dehydrated in ethanol, cleared in Clearene (Surgipath Europe Ltd.) and embedded in a wax block following conventional procedures, with the cut surfaces presented at the block face. Sections 3–5 μm thick were cut from each block face and stained with haematoxylin and eosin (H&E). Additional sections were cut from five wax blocks in each batch of 15 blocks derived from a single sheep. These sections were used in an *in situ* hybridization method to demonstrate the occurrence and distribution of apoptosis, as described below. All sections were examined and photographed using a Leica DM LB2 microscope with a Nikon Coolpix 5000 camera system.

### 2.3. In Situ Hybridization Method to Demonstrate Apoptosis

The TUNEL (TdT-mediated dUDP nick end labelling) *in situ* hybridization method, designed to specifically label endonuclease-induced DNA strand breaks associated with apoptosis, was carried out on sections of *in vivo* TCBZ-treated and untreated control TCBZ-S and TCBZ-R flukes using a commercially available kit (*In situ* Cell Death Detection POD kit, Cat.No. 1684817, Roche Diagnostics GmbH, Roche Molecular Biochemicals, Mannheim, Germany). The procedure used was based on that described in the Instruction Manual. Sections of the formalin-fixed flukes were de-waxed, rehydrated and equilibrated in 5 mM Tris-buffered saline (pH 7.6) (TBS). Endogenous peroxidases were blocked by incubation at room temperature in 0.5% (v/v) H2O2 in methanol for 20 min, following which the sections were again washed in TBS prior to incubation for 15 min at 37 °C in proteinase K (20 μg/mL in TBS) for antigen retrieval. Further TBS washing was followed by permeabilisation using 0.1% (v/v) Triton X in 0.1% (w/v) sodium citrate (2 min, 4 °C). After further TBS washing, 50 μL of TUNEL reaction mixture (containing TdT and fluorescein-labelled nucleotides) was applied to each section, for an incubation period of 60 min at 37 °C. Subsequent washing with 1% (v/v) bovine serum albumin (BSA) in TBS was followed by application of 50 μL Converter-POD (anti-fluorescein antibody conjugated with horse-radish peroxidase) for an incubation period of 30 min at 37 °C. After further washing with TBS + 1% BSA, diaminobenzidine (DAB) substrate for POD (Peroxidase Substrate kit DAB, Cat.No. SK-4100, Vector Laboratories Inc., Burlingame, CA, USA) was applied to the sections for 5–7 min. Final TBS washing of the sections was followed by counterstaining using Harris’s haematoxylin, and the sections were blued, dehydrated, cleared and mounted following conventional histological procedures. Negative control sections were prepared with each labelling batch. These sections were incubated with TUNEL reaction mixture from which the TdT was omitted. Positive controls were also included. To prepare them, TACS nuclease (TACS.XL DAB *In situ* Apoptosis Detection kit, Cat. No. 4828-30-DK, Trevigen Inc., Gaithersburg, MD, USA) was included in the TUNEL reaction mix. This exogenously applied nuclease generates DNA strand breaks in all the nuclei exposed in the tissue section.

### 2.4. Preparation and Examination of Stained Whole-Mounts

Flukes collected from field-infected sheep were fixed with 70% ethanol for at least 48h, and were subsequently incubated overnight at 22 °C in 0.1% catechol dissolved in 35% ethanol. Following a rinse in 35% ethanol they were transferred to borax carmine solution (2% sodium tetraborate with 1.5% carmine in 35% ethanol) and left to stain for 24 h. The flukes were then rinsed with 35% ethanol and transferred to 70% acidified ethanol (1 mL of concentrated HCl in 500 mL of 70% ethanol) to destain. The destaining process was carefully observed, with frequent changes of acidified ethanol, until the flukes just retained an overall pink colour. They were then rinsed in non-acidified 70% ethanol and transferred through two 3 h changes of absolute ethanol to xylene. The stained flukes were placed in xylene in a glass petri dish, and examined and measured using a low-power dissecting microscope with sub-stage illumination.

## 3. Results

### 3.1. Arrangement of the Gonads, Accessory Organs and Ducts

**Figure 1:**
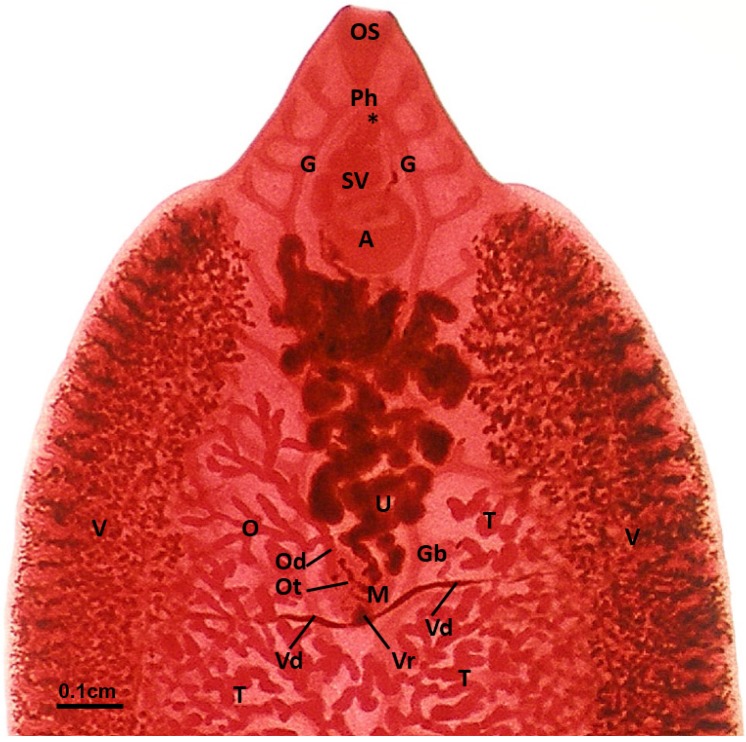
*Fasciola hepatica*: Carmine-stained whole mount of a fluke from an untreated field case of chronic fasciolosis in a sheep. Behind the oral sucker (**OS**) the two main caeca of the gut (**G**) bifurcate from the pharynx (**Ph**), and lateral branches of the gut (**Gb**) lie dorsal to the reproductive structures throughout the body. The position of the genital pore is marked (*), while the seminal vesicle (**SV**) is located anterior to the ventral sucker (or acetabulum, **A**). The two testes are highly branched (**T**), and these branches occupy the medial portion of the body, with the very numerous vitelline follicles (**V**) distributed in two broad lateral zones, merging at the posterior end of the fluke. The two main vitelline ducts (**Vd**), one from each side, supply the vitelline reservoir (**Vr**) at the caudal pole of the Mehlis’ gland complex (**M**). The single dendritic ovary (**O**), on the left side of the fluke, delivers oocytes via the oviduct (**Od**) to the ootype (**Ot**), where each oocyte associates with approximately 30 vitelline cells, and an egg-shell is formed around the cell mass. Completed eggs pass into the proximal coils of the uterus (**U**), and are stored there temporarily while being shifted forward towards the genital pore by the new eggs entering from behind.

The relative position of the main reproductive organs is evident in carmine-stained whole mounts (Figure 1). The two testes are much branched, one behind the other in the posterior half of the body. A vas deferens runs forward from each testis, and the two vasa deferentia join to form the seminal vesicle. The latter lies within the cirrus sac, and an ejaculatory duct leads forward through the protrusible cirrus, opening at the common genital pore which is medially situated between the oral and ventral suckers. Vitelline follicles fill up the space lateral and posterior to the testes. A single duct collecting vitelline cells from the vitelline tissue on each side of the body runs transversely to merge with the duct from the opposite side, forming the vitelline reservoir. The latter is located at the posterior margin of the Mehlis’ gland complex. From the vitelline reservoir, a common vitelline duct runs forward towards the ootype. The ovary is branched, situated in front of the testes and on the left (as viewed from the ventral surface). The oviduct, leading from the ovary, connects to the common vitelline duct, and the combined ovovitelline duct merges with the ootype. Each oocyte released from the oviduct associates with approximately 30 vitelline cells in the ootype. Under the influence of secretions from the Mehlis’ gland cells which surround the ootype, the precursor egg-shell proteins are released from each vitelline cell, and a contiguous, hyaline shell is moulded around the mass of vitelline cells and single ovum comprising each egg. As each egg is formed it passes forward into the proximal coils of the uterus. Spermatozoa, usually present in large numbers in the proximal uterus, achieve access to the oocyte before the egg-shell hardens.

### 3.2. Testis

**Figure 2:**
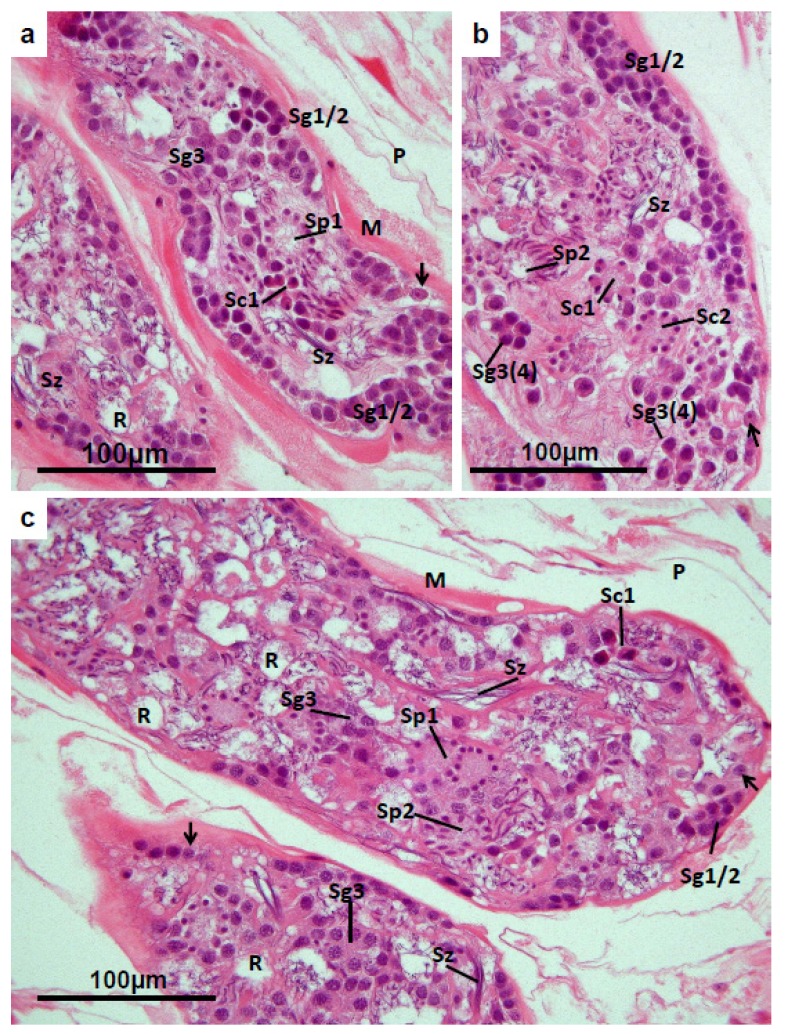
(**a,b**) *Fasciola hepatica*: Histology of the testis of a fluke from an untreated field case of chronic fasciolosis in a sheep. **(c)** Testis of a fluke from a TCBZ-resistant field case of fasciolosis, treated with TCBZ 21 days before collection. The profiles of the tubules lying in the parenchymal tissue (**P**) are demarked by an external layer of smooth muscle (**M**). Clusters of primary and secondary spermatogonia (**Sg1/2**) occur peripherally, while numerous tertiary spermatogonia (**Sg3**) are evident towards the core of the tubules, sometimes appearing as clusters of four cells (**Sg3(4)**, Figure 2b). Mitotic division in the tertiary spermatogonia, followed by incomplete cyto-segregation, result in the formation of 8-cell clusters of primary spermatocytes, connected by cytoplasmic bridges (**Sc1**). The cytoplasm of the primary spermatocytes is relatively eosinophilic. Primary spermatocytes undergo the first meiotic division, resulting in rosettes of 16 secondary spermatocytes, connected to a common cytoplasmic body (**Sc2**, Figure 2b). Completion of the second division of meiosis in each 16-cell rosette yields morula-like clusters of 32 early spermatids, each with a dense spherical nucleus, arranged around the periphery of a rather vacuolated common cytoplasmic body (**Sp1**, Figure 2a,c). Spermiogenesis proceeds with the elongation of the spermatid nuclei (**Sp2**, Figure 2b,c) which push out into the fluid matrix of the testis tubule, eventually detaching as rafts of maturing spermatozoa (**Sz**), and leaving residual vacuoles (**R**) which often contain a few twisted spermatid nuclei. At the periphery of each tubule, nuclei of the sustentacular tissue can often be recognised (unlabelled arrows). These are less dense than the nuclei of the neighbouring spermatogonia, and each features a prominent nucleolus.

**Figure 3:**
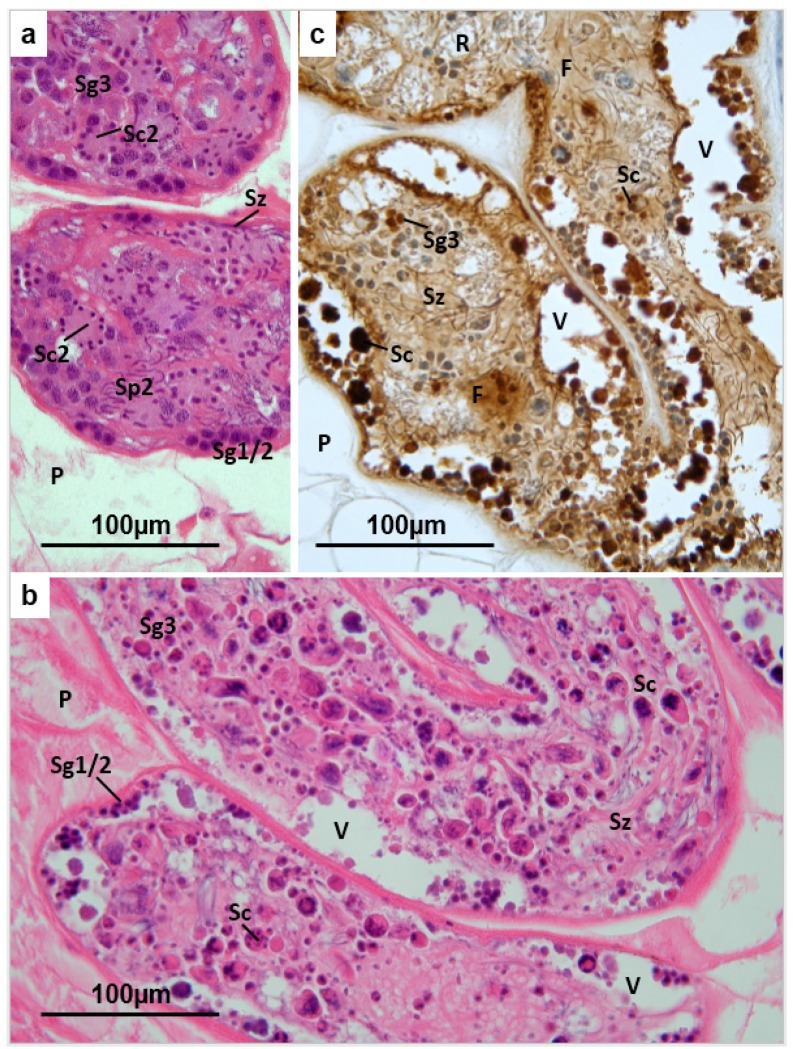
(**a**) *Fasciola hepatica*: Histology of the testis of a fluke from an untreated field case of chronic fasciolosis in a sheep. **Sg1/2** = primary and secondary spermatogonia; **Sg3** = tertiary spermatogonia; **Sc2** = secondary spermatocytes; **Sp2** = elongated spermatid nuclei; Sz = mature spermatozoa; (**b**) *Fasciola hepatica*: Histology of the testis of a TCBZ-sensitive fluke collected from a sheep two days after treatment with TCBZ. The tubules are rather depleted of cells, although clusters of spermatogonia (**Sg1/2**) are recognisable at the periphery. Spermatocytes (**Sc**) are represented by large rounded eosinophilic bodies containing irregular and fragmented masses of dense chromatin, while most of the smaller rounded eosinophilic cells with dense nuclei are likely to be tertiary spermatogonia (**Sg3**). Mature spermatozoa (**Sz**) are evident in the matrix of the tubules, while large vacuoles (**V**) containing cell debris, eosinophilic bodies and some spermatogonia are abundant at the periphery. **P** = parenchyma; (**c**) *Fasciola hepatica*: Testis of a TCBZ-sensitive fluke collected from a sheep two days after treatment with TCBZ. The section was treated by the TUNEL method to demonstrate apoptosis. The densest deposits of brown reaction product are associated with the large rounded bodies identified as spermatocytes (**Sc**) and with smaller rounded cells, probably tertiary spermatogonia (**Sg3**). The fluid matrix (**F**) of the testis tubules, which is less strongly labelled, surrounds maturing spermatozoa (**Sz**). Numerous apoptotic bodies are present in the peripheral vacuoles (**V**), while the residual vacuoles (**R**) present a relatively normal appearance. **P** = parenchyma.

A full account of the ultrastructural features of spermatogenesis and spermiogenesis in *F. hepatica* was given by Stitt and Fairweather (1990) [37]. In each and every normal sperm-producing fluke (whether untreated, or treated but TCBZ-resistant) the vast majority of the profiles of testis reveal cells representing all stages in the processes of spermatogenesis and spermiogenesis. Near the periphery of each profile, often in contact with the wall of the testis tubule, are primary and secondary spermatogonia that have basophilic cytoplasm and a high nucleo-cytoplasmic ratio (Figure 2a–c and Figure 3a). Towards the core of the tubules are located tertiary spermatogonia with larger, less intensely basophilic nuclei and more abundant, rather vesicular, cytoplasm (Figure 2a–c and Figure 3a). Whilst these sometimes appear in groups of four cells (Figure 2b), they lack the connecting cytoplasmic bridges that are characteristic of the cells in the later stages of spermatogenesis. Tertiary spermatogonia originate from primary spermatogonia by two successive mitotic divisions. A further mitotic division within these groups of tertiary spermatogonia gives rise to clusters of eight primary spermatocytes that are linked by cytoplasmic bridges. The primary spermatocytes exhibit dense peripheral nuclei and rather eosinophilic cytoplasm (Figure 2a–c). Sometimes, the chromosomes appear condensed and organised in a pattern consistent with meiotic metaphase or anaphase. The first meiotic division in the spermatocytes results in 16-cell clusters or rosettes of secondary spermatocytes. These are characterised by small dense spherical nuclei, each surrounded by a thin layer of eosinophilic cytoplasm, forming a tear-shaped protrusion from a common cytosome (Figure 2b and Figure 3a). The final meiotic division yields clusters of 32 spermatids located peripherally in a weakly staining cytosome. The spermatid nuclei are initially spheroidal or spindle-shaped (Figure 2a–c) but, with further development, become elongated, pushing out from the surface of the cytosome and sometimes appearing as a tangled cluster in a lucid vacuole (Figure 2b and Figure 3a). Elongation of each spermatid nucleus accompanies the development of an axonemal complex, and the motile, maturing spermatozoon is liberated from the residual cytosome to lie free in the lumen of the testis tubule (Figure 2a–c and Figure 3a). After the maturing spermatozoa have been liberated, the residual cytosome often remains as a spheroidal lucid vacuole within the testis tubule (Figure 2a–c), and may have a role in heterophagic breakdown of effete cell components, or in osmotic regulation within the tubule [38]. Maturing spermatozoa often appear in the testis profiles as rafts of thread-like basophilic nuclei in close parallel orientation (Figure 2a–c and Figure 3a). Maturing spermatozoa and developing spermatids are very abundant and conspicuous in most testis profiles in wild-type flukes. Heterophagic breakdown of abnormal or aborted developmental stages appears to be carried out by scavenging activity in the peripherally-located sustentacular tissue (Figure 2a–c). Profiles of mature spermatozoa, in large or small numbers, are generally seen in the coils of the uterus, amongst the shelled eggs (Figure 7a), and are particularly abundant in the proximal coils, just anterior to the ootype. The foregoing account of the histology of the testis is largely based on studies of flukes collected from naturally infected sheep and cattle, which produce normal-appearing spermatozoa, and readily engage in cross-insemination *in vivo*. However, in certain experimental isolates of TCBZ-S and TCBZ-R flukes, the histology of the testes differs from that of the wild-type flukes, consistent with defects in spermatogenesis or spermiogenesis, and in such cases cross-fertilization, and indeed self-fertilization, is not possible [11].

In TCBZ-S flukes (from field cases of TCBZ-S fasciolosis, and also from experimental TCBZ-S isolates) collected 24 h and subsequently after the host has been treated with TCBZ, there is a marked reduction in the number of cells present in the testis profiles. The consequent vacuolation represents an increase in “empty” or cell-free space within the tubules (Figure 3b). Some clusters of primary and secondary spermatogonia remain recognisable peripherally, while the numbers of tertiary spermatogonia are reduced in the centre of the profiles. Eosinophilic apoptotic spermatocytes are abundant, and many mononuclear cells, presumably spermatogonia, also display pyknotic nuclei and eosinophilic cytoplasm (Figure 3b). Later developmental stages such as secondary spermatocytes, spermatid clusters and mature spermatozoa are progressively reduced in number and integrity, and are often represented by pyknotic and karyorrhectic debris. After 72 h exposure in TCBZ-treated sheep, the testis profiles of TCBZ-S flukes are denuded of cellular contents and often appear shrunken. They contain a few eosinophilic bodies with irregular clumps of dense chromatin, fragments of dense chromatin and eosinophilic cell debris apparently lacking chromatin. In sheep treated 96 h previously with TCBZ, few TCBZ-S flukes remain in the liver. Some may be found in the gall bladder, having detached from the wall of the bile ducts. These flukes are dead and disintegrating. In order to verify or refute the contention that efficacy of TCBZ treatment is associated with apoptosis in the reproductive organs of flukes, histological sections of TCBZ-S and TCBZ-R flukes (from experimental isolates and field cases of fasciolosis) were subjected to the TdT mediated dUDP nick end labelling (TUNEL) *in situ* hybridization method. This commercially-available test is specifically designed to label endonuclease-induced DNA strand breaks associated with apoptosis [39]. It was found that in treated TCBZ-S flukes, strong positive labelling indicating apoptosis was associated with morphologically abnormal cells undergoing mitosis or meiosis in the testis. Background labelling in the positive testis sections indicated heterophagy of cell debris by the sustentacular tissue (Figure 3c). The histological changes that occur in the testis of TCBZ-S flukes collected from sheep treated for up to 72 h before slaughter with TCBZ or the related benzimidazole derivative, Compound alpha, were described in detail by Hanna *et al*. (2010) [31], and by McConville et al. (2010) [27].

### 3.3. Ovary

**Figure 4:**
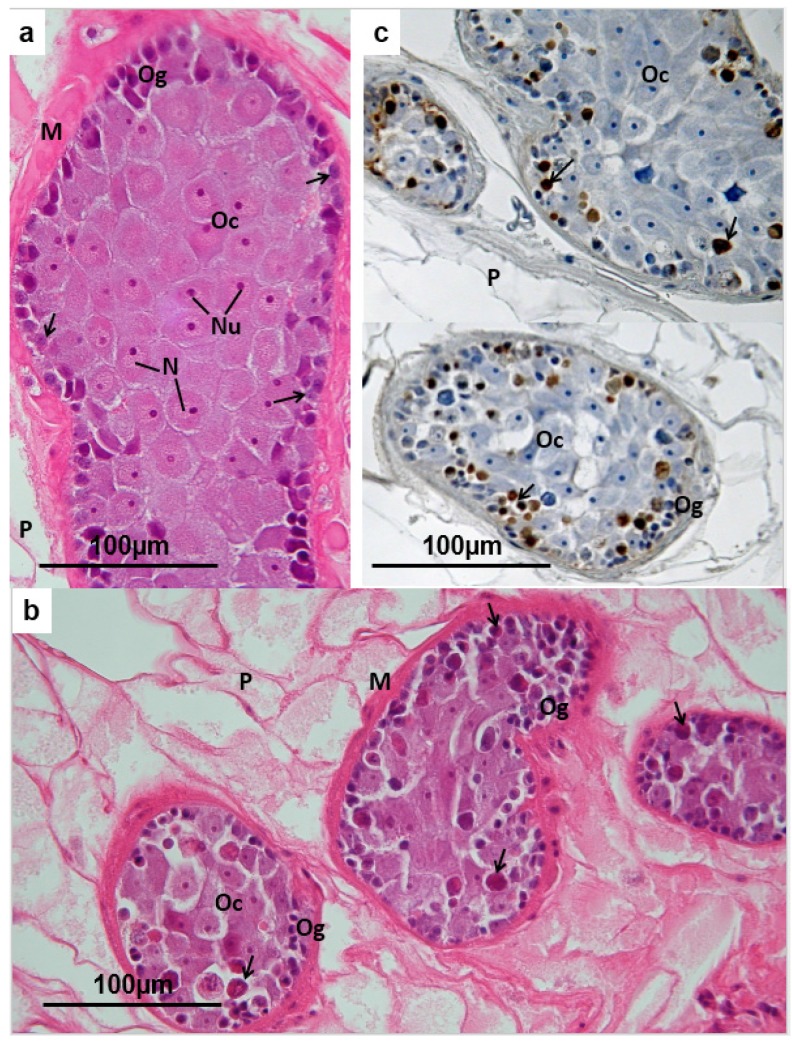
(**a**) *Fasciola hepatica*: Histology of the ovary of a fluke from an untreated field case of chronic fasciolosis in a sheep. The dendritic ovarian tubules are embedded in parenchyma (**P**), and enveloped by a thick layer of smooth muscle (**M**). Oogonia (**Og**), each containing a heterochromatic nucleus (within which a nucleolus is often visible) and densely-staining cytoplasm, are organised in a zone at the periphery of each tubule. They are interspersed with tissue featuring less densely staining, rather vacuolated cytoplasm and moderately heterochromatic nuclei, within which single prominent nucleoli are often seen (**unlabelled arrow**). The latter probably represent nurse cells. Deeper in the ovarian tubules large spherical or polygonal oocytes are packed together (**Oc**). The cytoplasm of each is basophilic, but less dense than that of the oogonia, and the periphery of each oocyte appears rather vesicular. The nucleus (**N**) of each oocyte is euchromatic, less dense that the cytoplasm, and features usually one prominent nucleolus (**Nu**). Towards the periphery of the tubules the oocytes sometimes appear rather elongated; (**b**) *Fasciola hepatica*: Histology of the ovary of a TCBZ-sensitive fluke collected from a sheep two days after treatment with TCBZ. The profiles of the ovarian tubules are shrunken and the contents are depleted of cellular content, appearing rather vacuolated. The peripheral zone of oogonia (**Og**) and nurse cells is discontinuous, with the less densely-staining oocytes (**Oc**) bulging out to the periphery in places. Many of the oocytes appear shrunken, having lost their plump spherical or polygonal outline, and there are numerous contracted, rounded eosinophilic cells in both the peripheral zone and in the core of each tubule. **M** = smooth muscle; **P** = parenchyma. (**c)** *Fasciola hepatica*: Ovary of a TCBZ-sensitive fluke collected from a sheep two days after treatment with TCBZ. The section was treated by the TUNEL method to demonstrate apoptosis. Dense deposits of brown reaction product label the rounded eosinophilic cells in the peripheral zone and core of each tubule. These apoptotic bodies represent oogonia (**Og**) and oocytes (**Oc**) respectively.

In TCBZ-R flukes, and in flukes collected from untreated sheep, profiles of each ovarian tubule display a relatively thick muscular wall, within which occurs a peripheral zone of small oogonia (each approximately 10 µm in diameter) and nurse cells. The nuclei of the latter are not readily distinguishable at the light microscope level (Figure 4a). The oogonia feature heterochromatic nuclei, sometimes in mitotic division, within which one or two nucleoli are often seen. The cytoplasm of each oogonium is represented by a thin basophilic layer enveloping the nucleus. Primary oocytes, derived by mitotic division of the oogonia, are seen to occupy the centre of each tubule, with little or no free space between cells (Figure 4a). Most are rounded or polygonal in shape, but in some profiles the oocytes appear elongated or rather irregular. The oocytes are larger than the oogonia (up to 25 µm diameter), and have abundant, generally less dense and sometimes slightly vesiculated cytoplasm. In certain profiles some or many of the oocytes have more dense, somewhat eosinophilic, cytoplasm. The nucleus of each oocyte is usually euchromatic, larger than that of the oogonia, and is often seen to bear a large single nucleolus. None of the nuclei exhibit progression beyond meiotic prophase. This description is consistent with the findings of Bjorkman and Thorsell (1964) and Gresson (1964) [40,41]. In TCBZ-S flukes from sheep treated with TCBZ 48 h or more before slaughter, the oogonia often appear shrunken and rounded, lying in a clear vacuole and featuring an unusually dense or pyknotic nucleus (Figure 4b). There is progressive loss of oogonia, with profiles of 48 h and 72 h-treated flukes often lacking this stage or displaying a discontinuous peripheral zone. Oocytes also are frequently seen to be shrunken, rounded and intensely eosinophilic, consistent with apoptosis. In sections labelled by the TUNEL method for endonuclease-generated DNA-strand breaks, generally there is intense labelling of the shrunken oogonia and oocytes in the periphery and core of the ovarian tubules (Figure 4c), confirming their apoptotic status. In the lumen of the tubules, the cells often lose contact, leaving irregular empty spaces. There is progressive loss of cellular content from the tubules, and a corresponding reduction in their diameter.

### 3.4. Vitelline Follicles

**Figure 5:**
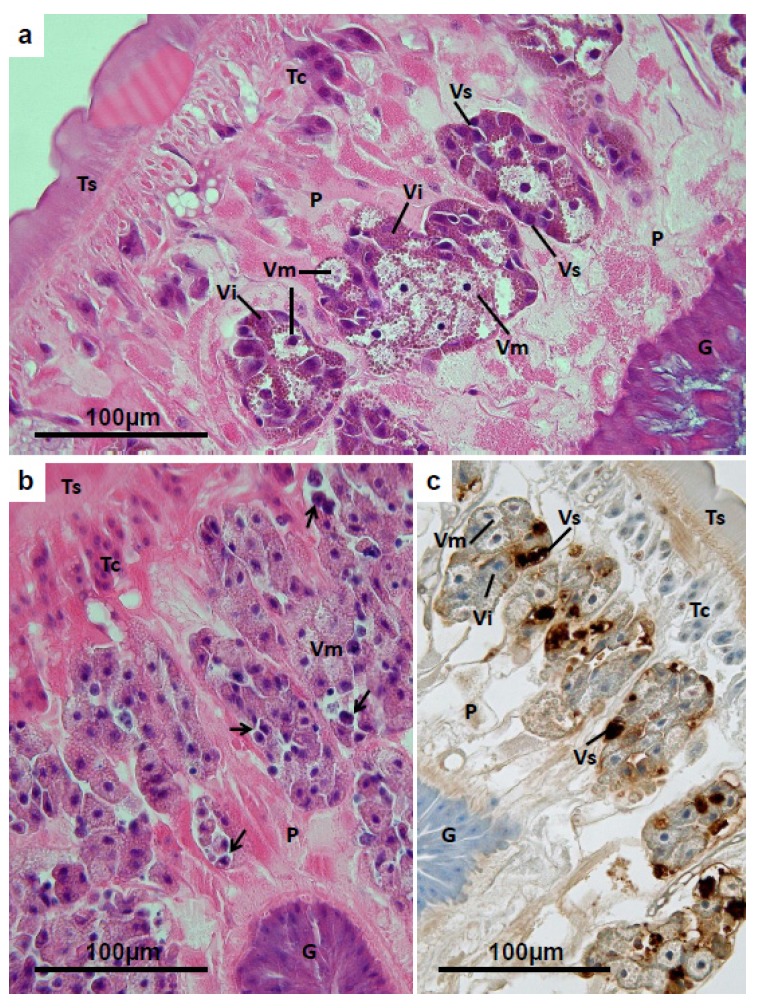
(**a**) *Fasciola hepatica*: Histology of vitelline follicles of a fluke from a TCBZ-resistant field case of fasciolosis, treated with TCBZ 21 days before collection. Within each follicle, stem cells and undifferentiated vitelline cells (**Vs**), featuring dense heterochromatic nuclei and basophilic cytoplasm, occupy a peripheral position. Intermediate vitelline cells (**Vi**) are characterised by the presence of brownish shell protein globules in the cytoplasm, while the mature vitelline cells (**Vm**) bulge with unstained glycogen, in addition to the shell protein globules, and are displaced towards the centre of the follicle, as they move to the efferent duct. **P** = parenchyma; **G** = gut; **Ts** = tegumental syncytium; **Tc** = tegumental cell bodies. (**b**) *Fasciola hepatica*: Histology of vitelline follicles of a TCBZ-sensitive fluke collected from a sheep two days after treatment with TCBZ. The vast majority of the vitelline cells remaining in the follicles are mature (**Vm**), containing unstained glycogen and shell protein globules. At the periphery of the follicles vacuolation is marked, and there are numerous rounded, isolated, dense eosinophilic bodies (unlabelled arrows), probably representing stem cells and undifferentiated vitelline cells. **P** = parenchyma; **G** = gut; **Ts** = tegumental syncytium; **Tc** = tegumental cell bodies; (**c**) Vitelline follicles of a TCBZ-sensitive fluke collected from a sheep two days after treatment with TCBZ. The section was treated by the TUNEL method to demonstrate apoptosis. The mature vitelline cells (**Vm**) and intermediate cells (**Vi**) are unlabelled, but dense deposits of brown reaction product are associated with the rounded eosinophilic bodies at the periphery of the follicles, indicating apoptosis in the stem cells and/or undifferentiated vitelline cells (**Vs**). **P** = parenchyma; **G** = gut; **Ts** = tegumental syncytium; **Tc** = tegumental cell bodies.

In the vitelline follicles of *F. hepatica*, various cell types reflecting different stages in the development and differentiation of vitelline cells are recognisable. Groups of approximately 30 mature vitelline cells associate with each oocyte during egg assembly in the ootype of the fluke. They contribute precursor protein to form the egg-shell, while the remaining cytoplasm, which is packed with glycogen laid down in the final stages of differentiation in the vitelline follicle, goes to provide an energy source for the embryonating egg. The ultrastructure of the vitelline follicle was described by Irwin and Threadgold (1970) [42]. Stem cells, which occur at the periphery of each follicle (Figure 5a), have basophilic cytoplasm lacking inclusions, and a large nucleo-cytoplasmic ratio. These divide by mitosis, giving rise to new stem cells to continue the somatic line, together with undifferentiated intermediate vitelline cells. The latter initiate active protein synthesis by a GER-Golgi-mediated process, accumulating yellowish refringent globules of shell precursor protein in the cytoplasm surrounding the enlarged nucleus (Figure 5a). A network of cytoplasmic nurse cell extensions surrounds, supports and nourishes the developing vitelline cells [42]. When the phase of protein synthesis is complete, each vitelline cell, now displaced towards the centre of the follicle by the pressure of developing cells beneath, undergoes a large expansion in cytoplasmic volume, due to the accumulation of glycogen and “yolk” bodies (autophagosomes). The shell protein globules come to be located in clusters at the periphery of each cell (Figure 5a). These mature vitelline cells are propelled along the vitelline ducts, which eventually merge to give rise to a single main transverse vitelline duct on each side of the fluke. These connect to the vitelline reservoir, subjacent to the ootype (Figure 1). In TCBZ-S flukes from hosts treated with TCBZ at least 24 h before slaughter, the vitelline follicles contain relatively more mature cells and intermediate cells and fewer stem cells than the vitelline follicles of untreated flukes (Figure 5b). Further, in many flukes the cell boundaries between the mature vitelline cells at the centre of each follicle are indistinct or lacking. The shell protein globules appear irregular in size and density, and are less clearly concentrated at the periphery of each cell. The stem cells often appear contracted within a clear vacuole and the nuclei are pyknotic or karyorrhectic, while the cytoplasm is eosinophilic (Figure 5b). In flukes collected 72 h and 96 h after the host has been treated with TCBZ, the vitelline follicles are progressively reduced in size, vacuolated, and contain mainly disrupted mature cells and cell debris. In TCBZ-treated sensitive flukes, the histological changes seen in the peripherally-located stem cells are consistent with apoptosis (rounding, isolation, pyknosis, cytoplasmic eosinophilia) and their apoptotic state has been confirmed by specifically labelling the endonuclease-induced DNA strand breaks using the TUNEL method [38] (Figure 5c).

### 3.5. Mehlis’ Gland

**Figure 6:**
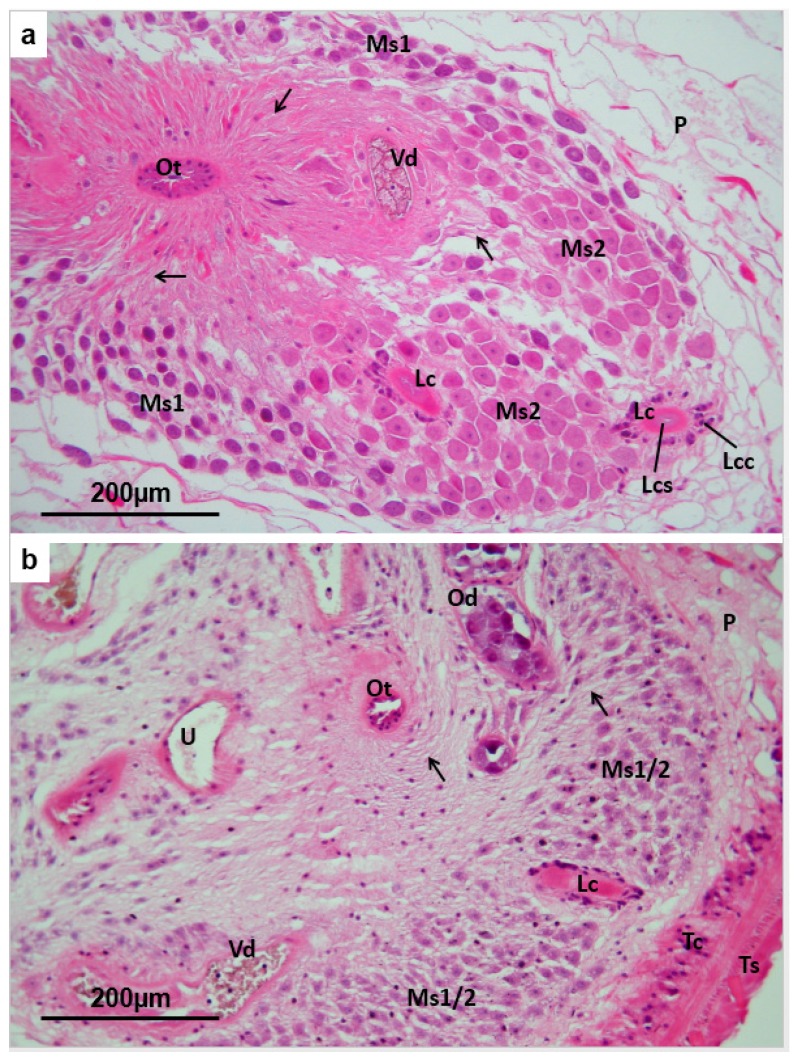
(**a**) *Fasciola hepatica*: Histology of the Mehlis’ gland of a fluke from an untreated field case of chronic fasciolosis in a sheep. The organ comprises elongated S1 secretory cells (**Ms1**) with basophilic cytoplasm, which are mainly located towards the periphery of the complex, ventrally and laterally, and plump S2 secretory cells (**Ms2**) with paler eosinophilic cytoplasm, mainly located centrally and dorsally. Elongated projections (**unlabelled arrows**) from both types of cell converge on the ootype (**Ot**) and contain respectively basophilic and eosinophilic secretory material. Laurer’s canal (**Lc**) tracks from the dorsal surface of the fluke to the ootype. Its lining (**Lcs**) is continuous with the tegumental syncytium, while tegumental cell bodies (**Lcc**) lie deeper. **P** = parenchyma; (**b**) *Fasciola hepatica*: Histology of the Mehlis’ gland of a TCBZ-sensitive fluke collected from a sheep two days after treatment with TCBZ. The cytoplasm of the S1 and S2 secretory cells (**Ms1/2**) is shrunken, pale and vesiculated, making distinction of the two cell types difficult. The elongated tubules (**unlabelled arrows**) connecting the secretory cells to the ootype (**Ot**) are also vesiculated and lack content. Profiles of the oviduct (**Od**) contain rounded eosinophilic oocytes, while the common vitelline duct (**Vd**) contains disintegrating vitelline cells. **Lc** = Laurer’s canal; **P** = parenchyma; **U** = proximal uterus; **Ts** = tegumental syncytium; **Tc** = tegumental cell bodies.

In *F. hepatica*, the Mehlis’ gland comprises two types of cell, S1 and S2, arranged radially around the ootype, but lying at different distances from it and connected to the ootype by cytoplasmic extensions, along which the secretory products are channelled [43]. Both types of cell are distributed in a supporting parenchymal matrix (Figure 6a). The S1 cells, which tend to lie further from the ootype than the larger S2 cells, have rather dense basophilic cytoplasm and produce elongated basophilic secretory bodies which are often evident in the cytoplasmic extensions. The S2 cells have a less dense, rather vesicular cytoplasm and produce eosinophilic secretions (Figure 6a). In TCBZ-S flukes collected from hosts that have been treated with TCBZ, the Mehlis’ gland cells of both types show shrinkage and vacuolation of the cytoplasm (Figure 6b), which is progressively more marked in the 72 h and 96 h-treated flukes. Vacuolation is also evident in the cytoplasmic extensions, and in the supporting parenchymal matrix. The secretions from the S1 and S2 cells are absent in the treated flukes, and due to the vacuolation of the cell bodies and reduction of basophilic staining in the S1 cells, the two types of Mehlis’ gland cell become almost indistinguishable (Figure 6b). Since apoptotic cells are not present in the Mehlis’ gland of TCBZ-S treated flukes, there is no positive labelling with the TUNEL method, indicating the absence of endonuclease-induced DNA strand breaks.

### 3.6. Uterus

**Figure 7:**
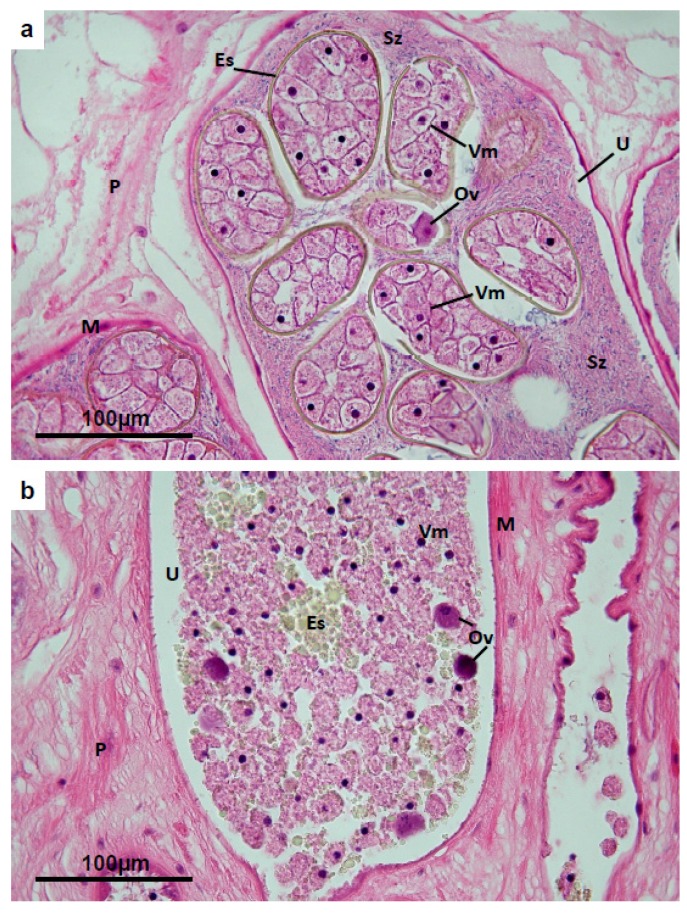
(**a**) *Fasciola hepatica*: Histology of the uterus of a fluke from a TCBZ-resistant field case of fasciolosis, treated with TCBZ 21 days before collection. The uterine tube is invested by smooth muscle (**M**), and surrounded by parenchyma (**P**). Typically, profiles of the uterus are packed with fully-formed eggs, each encapsulated in a contiguous, hyaline yellowish egg-shell (**Es**) and containing a single ovum (**Ov**) together with approximately 30 mature vitelline cells (**Vm**). Each of the latter contains pale-staining cytoplasm with glycogen and a single dense nucleus, but no shell-protein globules. Numerous mature spermatozoa (**Sz**) are present in the uterine lumen; (**b**) *Fasciola hepatica*: Histology of the uterus of a TCBZ-sensitive fluke collected from a sheep two days after treatment with TCBZ. No intact eggs remain in the uterine lumen (**U**), which is largely occupied by disintegrating vitelline cells (**Vm**), irregular hyaline masses of egg-shell protein (**Es**), and occasional ova (**Ov**). **M** = uterine wall muscle; **P** = parenchyma.

In flukes freshly collected from untreated host animals (and in TCBZ-R flukes taken from TCBZ-treated hosts), the uterus, which coils in an anterior direction from the ootype to the genital pore, is generally found to be turgidly packed with well-formed eggs. Each egg is invested by a yellow-coloured hyaline shell approximately 2 µm thick, and contains numerous glycogen-filled vitelline cells together with a single ovum characterised by dense, rather basophilic cytoplasm (Figure 7a). In most flukes from field infections, and from normal sperm-producing experimental isolates such as the TCBZ-R Sligo isolate (Section 2.1.1) and the TCBZ-S isolate grown in rats (Section 2.1.3), spermatozoa are abundant in the uterus, especially in the proximal coils (*i.e.*, those closest to the ootype) [11]. In TCBZ-S flukes from sheep treated 24 h or more previously with TCBZ, the uterus contains few or no shelled eggs, but the proximal coils of the organ contain loose vitelline cells lacking shell protein globules in the cytoplasm, occasional free ova, and irregular masses of yellow-coloured hyaline shell protein (Figure 7b). In TCBZ-S flukes collected 72 and 96 h from TCBZ-treated hosts, the uterine coils display only sparse cellular and hyaline protein content and appear contracted. Flukes from animals treated 24 h prior to slaughter sometimes have normal shelled eggs remaining in the distal coils of the uterus (*i.e.*, nearest the genital pore), while the proximal coils are occupied by unshelled cell clusters and irregular hyaline masses, as described above. Apoptotic cells are not present in the uterus of TCBZ-S treated flukes, and consequently there is no positive labelling of endonuclease-induced DNA strand breaks.

## 4. Discussion

A number of investigations have been carried out on the morphological changes induced in *F. hepatica* by exposure to TCBZ and its active metabolites. These studies, reviewed by Fairweather and Boray (1999) [12] and Fairweather (2009) [16], have mainly involved the use of Scanning and Transmission Electron Microscopy on flukes exposed *in vitro* to physiological concentrations of the molecules in question. They have yielded much useful information relating to the possible mechanisms of drug action on the parasite. Most of the changes described are consistent with the known anti-tubulin effects of benzimidazoles, but, in addition, inhibition of protein synthesis has also been evoked as a possible mode of action [44]. 

The histological features of the testis in TCBZ-R flukes and in TCBZ-S flukes from untreated hosts described here were found to be broadly consistent with the ultrastructural findings of Stitt and Fairweather (1990) [37]. In the testis tubules of TCBZ-S flukes from sheep treated with TCBZ there was a marked and progressive increase in the proportion of cells that displayed degenerative features such as multiple nuclei, pyknotic or karyorrhectic nuclei, eosinophilic cytoplasm and rounded profiles. These changes are consistent with apoptosis, the fundamental phenomenon of “internally-programmed cell death” that occurs in all metazoans, and serves to eliminate cells that are irreparably damaged, particularly if that damage affects the DNA [45]. Many of the cells showing morphological changes associated with apoptosis in the testis of TCBZ-S flukes exposed to metabolites of the drug *in vivo*, also gave a strong positive labelling with the TUNEL method. This confirms the occurrence of endonuclease-induced DNA strand breaks, a marker of apoptosis, in these abnormal cells, and supports the concept that TCBZ activity may target spindle formation in dividing cells, triggering the cascade of events that leads to apoptosis. Recently, it has been shown that sustentacular tissue is located in the peripheral zone of the testis tubules in *F. hepatica* [38]. A primary function of this tissue appears to be the scavenging of effete cells and cytoplasmic debris, as well as recycling of useful molecules. This is carried out by a process of heterophagic digestion using lysosomal enzymes generated in the cytoplasm of the sustentacular tissue. Presumably the sustentacular tissue may have a role in scavenging cells damaged by TCBZ action.

Progressively fewer cells were present in the testis tubules of TCBZ-S flukes from treated sheep than in those of flukes from untreated sheep, with a corresponding increase in “empty” space. This probably followed from inhibition of mitotic division in the primary, secondary and tertiary spermatogonia by the metabolites of TCBZ. The activity of the benzimidazole group of anthelmintics, to which TCBZ belongs, is believed to lie in their selective ability to bind β-tubulin, thus inhibiting microtubule-mediated processes such as spindle formation during mitosis [46]. Fairweather (2005, 2009) [13,16] has reviewed the data linking the *in vitro* effects of TCBZ metabolites on *F. hepatica* with those of established microtubule inhibitors such as tubulozole-C. As observed in the present study, with inhibition of mitosis, it is likely that the testis tubules become progressively depopulated as the degenerating cellular contents exit through the vasa deferentia and genital pore. As expected, the histological features of the testis in TCBZ-R flukes were unaffected by anthelmintic treatment of the host, consistent with the findings of McConville *et al.* (2009a) [6].

The successful formation of a normal shelled egg in the ootype of *F. hepatica* represents the culmination of a complex sequence of cytokinetic and cytological events involving cells originating from the ovary and the vitelline follicles. There is also input of proteinaceous secretory material, including lipoproteins, from the Mehlis’ gland cells that envelop the ootype [12,47,48]. The co-ordination of cell movements and release of secretory products in the ootype region is achieved by neural and neurosecretory influences. While the neuroanatomy of the region has been mapped in elegant studies by Fairweather *et al*. (1987) [49] and Magee *et al*. (1989) [50], the exact mechanism awaits clarification. Clearly, drug-induced defects in the function and output of the ovary, vitelline system and Mehlis’ gland, as well as the neural/peptidergic mechanisms controlling the activity of the ootype [12,48], are likely to be reflected in the appearance and integrity of the eggs emerging from the ootype and entering the proximal coils of the uterus. The histological features of the uterus and its contents therefore provide an early and sensitive indication of the emergence of malfunctions throughout the female reproductive tract. In each and every TCBZ-S fluke exposed to TCBZ metabolites *in vivo* for 48 h or 72 h, no normal eggs were observed in the uterus. Instead, there were free vitelline cells (lacking peripheral shell globule clusters), free ova and irregular masses of coagulated shell protein material in the uterine lumen. In the ootype, the mechanism involved in laying down a coherent uniform layer of shell protein material to form a shell around each group of de-granulated vitelline cells, with their single associated ovum, had clearly failed. The underlying reason for this failure lay perhaps in abnormalities in the shell protein precursors, abnormalities in the Mehlis’ gland secretions or in disorganisation of the mechanical activity of the ootype. The onset of abnormal ovigenesis in these individuals occurs within 24h of administration of TCBZ to the host [10]. In flukes exposed for 72 h in TCBZ-treated hosts and in degenerating flukes from animals treated 96 h before slaughter with TCBZ, the uterine coils were largely empty. This reflected, not only loss of the content via the genital pore, but also cessation of the movement of vitelline cells and ova into the ootype. TCBZ-R flukes retain a full complement of maturing eggs in the uterus, reflecting uninterrupted ovigenesis and integrity of all components of the female reproductive tract, throughout the treatment period.

Within each vitelline follicle of *F. hepatica*, mitotic activity in the stem cells at the periphery provides a continuous supply of cells destined for shell protein production and glycogen storage. At the same time it ensures continuity of the stem cell line. Maturing vitelline cells differentiate progressively towards the core of the follicle, undergoing a phase of protein synthesis, during which shell globule clusters accumulate in the cytoplasm. Finally they reorganise as intracellular glycogen storage sites) [42,51]. As in the testis tubules, disruption of mitosis by the action of TCBZ metabolites *in vivo* results in apoptosis of stem cells, yielding discrete labelling of individual cells at the periphery of the follicles in TUNEL preparations. The break in supply of immature vitelline cells leads to progressive disappearance of early and intermediate stages in the follicles, as pre-formed cells mature. Production of shell protein globules and deposition of glycogen apparently continues in the presence of TCBZ metabolites *in vivo*. However, increasing numbers of mature vitelline cells within the follicles show evidence of disruption, with release and coalescence of the shell protein globules. The material so released assumes an unusually dark and condensed appearance. This possibly suggests that the enzyme-mediated chemical processes involved with quinone-tanning of the egg-shell [12,48] have been initiated prematurely. The role of TCBZ metabolites in the apparent disintegration of mature vitelline cells is unclear, although failure in onward propulsion through the vitelline ducts may be a factor. *In vitro* and *in vivo* treatment of *F. hepatica* with TCBZ metabolites has been shown to disrupt muscle fibres [20,21,24,25]. Therefore, lack of muscle tone in the duct walls, coupled with failure of cell pressure following inhibition of mitosis at the periphery of the follicles, may delay the exit of mature vitelline cells and result in partial disintegration. Consequently, precocious tanning of the shell protein material proceeds. As expected, TCBZ-R flukes taken from TCBZ- treated hosts showed no histological abnormalities in the vitelline tissues. 

In contrast to the situation in the testis, vitelline follicles and ovary of *F. hepatica*, no mitotic or meiotic activity occurs in the Mehlis’ gland complex. Therefore, it is not surprising that the histological features associated with anti-tubulin action in TCB- treated hosts, namely apoptosis and progressive decline in cell population, are not seen in the Mehlis’ gland. However, the apparent shrinkage of the S1 and S2 cells and loss of secretory material from the cytoplasm and connecting tubules is consistent with decline or cessation of the normal protein or lipoprotein secretory activity. It is also possible that the disruption of synthetic activity in the Mehlis’ gland complex is due to failure of energy metabolism. Fairweather and Boray (1999) [12] have reviewed evidence that TCBZ metabolites are capable of inhibiting protein synthesis in *F. hepatica*, and that benzimidazoles may be implicated in uncoupling oxidative phosphorylation. However, bearing in mind the well-established anti-tubulin activity of anthelmintics in this class, general disruption of metabolism in the Mehlis’ gland cells, and indeed ultrastructural disorganisation of organelles such as the GER, mitochondria and Golgi complexes leading to cytoplasmic vacuolation, may well be secondary to failure of microtubule-mediated intracellular processes.

The viscous alkaline lipoprotein secretions from the Mehlis’ gland cells are believed to play a key role in the uniform spreading and coalescence of the shell precursor proteins during egg-shell formation in the ootype. This is by virtue of the interface they form with the less viscous acidic fluid surrounding the vitelline cells [12,48]. Alteration in the physicochemical properties of Mehlis’ gland secretion might possibly reflect preferential accumulation of the hydrophobic molecules of the anthelmintic metabolites in the lipid component. Clearly, even minor alterations in the physicochemical constitution of the Mehlis’ gland secretions would be liable to have a major impact on proper formation of the egg-shell. Indeed, the disruption of this process, with the appearance of loose vitelline cells and aberrant coagulated masses of shell protein, was a prominent histological feature in the uterus of TCBZ-S flukes from the earliest stages of exposure to TCBZ *in vivo*.

The main histological changes seen in the ovaries of TCBZ-S flukes from hosts treated with TCBZ before slaughter, included progressive decline in cell population (with resulting constriction of the ovarian tubules and appearance of vacuoles). Furthermore, there was development of apoptotic changes in the remaining cell population. The TUNEL labelling pattern in the ovarian tubules of TCBZ-treated sensitive flukes probably reflects apoptosis in oogonia attempting to initiate mitosis, and oocytes in the initial stages of the first meiotic division. Primary oocytes move out of the ovary at the end of prophase of the first meiotic division, completing their development in the proximal coils of the uterus [41]. Failure of mitosis in the oogonia, resultant decline in cell output and initiation of apoptosis are consistent with defective spindle formation, initiated by TCBZ action. No histological abnormalities developed in the ovaries of the TCBZ-R flukes collected from treated sheep, as was the case with all the other reproductive structures. 

While the contribution of electron microscopical techniques to the analysis of drug-induced lesions in *F. hepatica* is well established, conventional histopathological methods have not often been employed. This is despite their potential value in screening simultaneously all tissues in representative samples of flukes exposed to anthelmintic drugs *in vivo* or *in vitro*. The present investigation seeks to provide a description and overview of lesions that develop in the reproductive organs of TCBZ-S liver flukes in host animals treated with TCBZ. Given the ease and rapidity with which large numbers of liver flukes can be processed for histology, it is possible to apply a semi-quantitative scoring system. This enables the overall severity of drug induced abnormality to be calculated for each treatment regimen [31]. A more rigorous quantitative approach might be achieved by the use of an “image analysis” programme. This would enable statistical comparison of results, and might prove particularly interesting in cases where TCBZ resistance was partial. It might also have a role in comparative assessment of the activities of putative anthelmintic molecules in field trials. The utility of histological analysis in aiding the choice of appropriate anthelmintic for the control of fasciolosis in field situations has been demonstrated. A recent study compared the relative efficacies of TCBZ, nitroxynil and closantel in reducing the fluke burden in sheep on farms throughout Northern Ireland [30]. This study also exemplified the use of histology as one of several complementary methods (FECRT; CRT; fluke histology; comparative anthelmintic efficacy testing) for the confirmation of a diagnosis of fluke drug resistance, as recommended by Fairweather (2011) [52].
